# Supplementary material for: The archives are half-empty: an assessment of the availability of microbial community sequencing data
Source: Commun Biol. 2020 Aug 28;3:474. doi: 10.1038/s42003-020-01204-9 (PMC7455719; doi:10.1038/s42003-020-01204-9)
Supplement: Supplementary file 2 — Description of Additional Supplementary Files [file 42003_2020_1204_MOESM2_ESM.pdf]

Description of Additional Supplementary Files

File Name: **Supplementary Data 1**
Description: The raw set of data outputted by our custom text-parsing algorithm. Each accession number occuppies one row, and additional columns include further study metadata (i.e., 16Sness=was "16S mentioned?", gene region=v1-v9)

File Name: **Supplementary Data 2**
Description: Here, 150 articles which mentioned 16S but for which no accession number or alternative database was detected were manually checked to ensure the parsing algorithm was working properly

File Name: **Supplementary Data 3**
Description: Articles which mentioned "16S", "515" and "806" are included here, and separated into articles which include INSDC-compliant accession numbers, and articles which reported using alternative databases Qiita, MG-RAST, or figshare

File Name: **Supplementary Data 4**
Description: This is the preliminary google search (via Publish or Perish) that was used to select the 17 specialist journals. All publications in these journals were then downloaded for text parsing

File Name: **Supplementary Data 5**
Description: Here, we compare the number of studies using 16S that we found with the number of studies citing QIIME or MOTHUR in the same period of time

File Name: **Supplementary Data 6**
Description: These are the raw results of our study, and include information on data, metadata, and data access for all 16S studies with INSDC-compliant accession numbers found

File Name: **Supplementary Data 7**
Description: Similar to sheet 6, here we summarize all findings for the V3V4 subset, including more detailed information on data access.
