# Supplementary material for: The archives are half-empty: an assessment of the availability of microbial community sequencing data
Source: Commun Biol. 2020 Aug 28;3:474. doi: 10.1038/s42003-020-01204-9 (PMC7455719; doi:10.1038/s42003-020-01204-9)
Supplement: Supplementary file 4 — Reporting Summary [file 42003_2020_1204_MOESM4_ESM.pdf]

nature research

Corresponding author(s): Stephanie D. Jurburg

Last updated by author(s): Jul 18, 2020

Reporting Summary

Nature Research wishes to improve the reproducibility of the work that we publish. This form provides structure for consistency and transparency in reporting. For further information on Nature Research policies, see our Editorial Policies and the Editorial Policy Checklist.

Statistics

For all statistical analyses, confirm that the following items are present in the figure legend, table legend, main text, or Methods section.

n/a	Confirmed	
☐	☒	The exact sample size (n) for each experimental group/condition, given as a discrete number and unit of measurement
☐	☒	A statement on whether measurements were taken from distinct samples or whether the same sample was measured repeatedly
☐	☒	The statistical test(s) used AND whether they are one- or two-sided<br>*Only common tests should be described solely by name; describe more complex techniques in the Methods section.*
☒	☐	A description of all covariates tested
☒	☐	A description of any assumptions or corrections, such as tests of normality and adjustment for multiple comparisons
☐	☒	A full description of the statistical parameters including central tendency (e.g. means) or other basic estimates (e.g. regression coefficient) AND variation (e.g. standard deviation) or associated estimates of uncertainty (e.g. confidence intervals)
☐	☒	For null hypothesis testing, the test statistic (e.g. F, t, r) with confidence intervals, effect sizes, degrees of freedom and P value noted<br>*Give P values as exact values whenever suitable.*
☒	☐	For Bayesian analysis, information on the choice of priors and Markov chain Monte Carlo settings
☒	☐	For hierarchical and complex designs, identification of the appropriate level for tests and full reporting of outcomes
☒	☐	Estimates of effect sizes (e.g. Cohen's d, Pearson's r), indicating how they were calculated

Our web collection on statistics for biologists contains articles on many of the points above.

Software and code

Policy information about availability of computer code

Data collection	Publish or Perish, Citavi, Grobid (https://github.com/kermitt2/grobid/), custom text partsing code (https://github.com/komax/teitocsv), cutadapt v1.18, FastQC v0.11.3. All codes used during data collection have been provided to the reviewers, and have been made available in Zenodo https://zenodo.org/record/3953314#.Xxam0igzaUk and http://doi.org/10.5281/zenodo.3953307
Data analysis	All data analyses were performed in R 3.6.1 with the tidyverse packages (particularly ggplot2 and dplyr). All codes used during analysis have been made available in Zenodo https://zenodo.org/record/3953314#.Xxam0igzaUk and http://doi.org/10.5281/zenodo.3953307

For manuscripts utilizing custom algorithms or software that are central to the research but not yet described in published literature, software must be made available to editors and reviewers. We strongly encourage code deposition in a community repository (e.g. GitHub). See the Nature Research guidelines for submitting code & software for further information.

Data

Policy information about availability of data

All manuscripts must include a data availability statement. This statement should provide the following information, where applicable:

- Accession codes, unique identifiers, or web links for publicly available datasets
- A list of figures that have associated raw data
- A description of any restrictions on data availability

The full dataset has been made available in Zenodo http://doi.org/10.5281/zenodo.3953307

Field-specific reporting

Please select the one below that is the best fit for your research. If you are not sure, read the appropriate sections before making your selection.

☐ Life sciences ☐ Behavioural & social sciences ☒ Ecological, evolutionary & environmental sciences

For a reference copy of the document with all sections, see nature.com/documents/nr-reporting-summary-flat.pdf

Ecological, evolutionary & environmental sciences study design

All studies must disclose on these points even when the disclosure is negative.

Study description	We selected the 17 journals which published high quality microbial ecology research, and parsed all of the articles published by these journals between January 2015 and February 2019 (inclusive), to select the 2015 articles and corresponding accession numbers for the studies which performed 16S rRNA amplicon sequencing.
Research sample	The sample is all studies from the 17 selected journals, published between January 2015 and February 2019 (inclusive). From these, we selected the articles and corresponding INSDC-compliant accession numbers for the studies which performed 16S rRNA amplicon sequencing. Sequencing data and metadata was obtained from INSDC-compliant databases (SRA, DDJ, and EBI)
Sampling strategy	All qualifying studies within the 17 journals selected, were included in the study. We used a custom-built parsing algorithm to digitally 'read' these ~30,000 articles to find those that had performed 16S rRNA amplicon sequencing. We excluded non-specialist journals as well as Biorxiv manuscripts, as we wanted to sample the articles which were published in microbiome-heavy journals, which might have stronger data archiving policies in place.
Data collection	Journals were selected by SDJ using a preliminary Publish or Perish-mediated Google Scholar search. They were parsed by MK, and the accession numbers were further examined by AHB.
Timing and spatial scale	All of the articles published by the 17 selected journals between January 2015 and February 2019 (inclusive) were parsed.
Data exclusions	We excluded articles published in generalist journals (i.e., PLOS One, Scientific Reports) as well as manuscripts in Biorxiv, as we aimed to focus on articles which had gone through the publication process in journals with microbiome-specific data archiving practices.
Reproducibility	We compared the number of articles which qualified for our study to the number of articles citing popular bioinformatics platforms for 16S rRNA gene amplicon sequencing (QIIME and USEARCH) in a Web of Science search. This is available in our supplementary data.
Randomization	N/A
Blinding	Blinding was not relevant to our study, as we used a parsing algorithm to select our data. To confirm the accuracy of our algorithm, we read 150 randomly selected articles which were not selected and confirmed that they had not performed 16S rRNA amplicon sequencing, that they hadn't included their sequence data in the supplementary materials, and that they hadn't been deposited to other undetected repositories.

Did the study involve field work? ☐ Yes ☒ No

Reporting for specific materials, systems and methods

We require information from authors about some types of materials, experimental systems and methods used in many studies. Here, indicate whether each material, system or method listed is relevant to your study. If you are not sure if a list item applies to your research, read the appropriate section before selecting a response.

Materials & experimental systems

n/a	Involved in the study	
☒	☐	Antibodies
☒	☐	Eukaryotic cell lines
☒	☐	Palaeontology and archaeology
☒	☐	Animals and other organisms
☒	☐	Human research participants
☒	☐	Clinical data
☒	☐	Dual use research of concern

Methods

n/a	Involved in the study	
☒	☐	ChIP-seq
☒	☐	Flow cytometry
☒	☐	MRI-based neuroimaging
